# A Systematic Review of Visualization Techniques and Analysis Tools for Eye-Tracking in 3D Environments

**DOI:** 10.3389/fnrgo.2022.910019

**Published:** 2022-07-13

**Authors:** Veronica Sundstedt, Valeria Garro

**Affiliations:** Department of Computer Science, Blekinge Institute of Technology, Karlskrona, Sweden

**Keywords:** 3D visualization, eye tracking, gaze, visual attention, virtual environments, systematic literature review

## Abstract

This systematic literature review presents an update on developments in 3D visualization techniques and analysis tools for eye movement data in 3D environments. With the introduction of affordable and non-intrusive eye-tracking solutions to the mass market, access to users' gaze is now increasingly possible. As a result, the adoption of eye-tracking in virtual environments using head-mounted displays is expected to increase since the trend is to incorporate gaze tracking as part of new technical solutions. The systematic literature review presented in this paper was conducted using the Scopus database (using the period 2017 to 17th of May 2022), which after analysis, resulted in the inclusion of 15 recent publications with relevance in eye-tracking visualization techniques for 3D virtual scenes. First, this paper briefly describes the foundations of eye-tracking and traditional 2D visualization techniques. As background, we also list earlier 3D eye-tracking visualization techniques identified in a previous review. Next, the systematic literature review presents the method used to acquire the included papers and a description of these in terms of eye-tracking technology, observed stimuli, application context, and type of 3D gaze visualization techniques. We then discuss the overall findings, including opportunities, challenges, trends, and present ideas for future directions. Overall the results show that eye-tracking in immersive virtual environments is on the rise and that more research and developments are needed to create novel and improved technical solutions for 3D gaze analysis.

## 1. Introduction

Eye-tracking technology provides the recording of eye movements of a user to determine the gaze direction and capture where he or she is looking during an interval of time. In recent years, there has been an increase in using portable eye-tracking solutions, such as glasses, for gathering gaze data from people observing their environment. Over the years, it has been common to use eye-tracking to analyze eye movements on traditional screens using 2D stimuli. Eye-movement data can also be used to analyze human behavior in 3D virtual environments (VEs) while using immersive head-mounted displays (HMDs). The eye movements from an observer indicate where overt visual attention is focused (Carrasco, 2011). Eye-tracking information can also be used in real-time to create gaze-controlled interfaces.

Screen-based eye-tracking for analyzing stimuli such as images, video, and VEs has commonly been used in the last decades. It is only recently that eye-tracking has started to appear in virtual reality (VR) or mixed reality (MR) to a larger extent. Several HMDs that incorporated eye-tracking, such as HTC Vive Pro Eye, FOVE 0, and Varjo VR-3, are available on the market and have been mass-produced. There are also solutions that allow adding binocular eye-tracking to existing headsets, for example, a binocular add-on by Pupil Labs. Eye-tracking has increasingly started to be used in real-time visual applications, including games (Sundstedt, 2012), simulators (Groner and Kasneci, 2021), and visual analytics (Burch, 2021). These applications generate more data than ever, and this trend is likely to continue. Eye-tracking and visual attention focus have also been exploited in HMDs to render some areas where the observer is looking in higher quality. This selective rendering process is also referred to as foveated rendering (Patney et al., 2016).

Game analytics (Nasr et al., 2013) is also a field that has grown significantly over the past decade. Game developers and designers have initially used game analytics to improve game design, evaluate performance, or study player behaviors. Players have adopted it to analyze matches of their own and others in novel ways. Recent video games generate a large amount of data to be analyzed, and with emerging technologies, such as VR games exploiting eye-tracking in HMDs, further data related to face, head, and body movement are gathered. Also, the area of eSports is growing rapidly, and here eye-tracking can be used to explore cognitive load and performance (Dahl et al., 2021). Another example is to explore observers' visual attention in relation to eSport advertisements (Seo et al., 2018). There is also an increased potential for HMD devices, which incorporate eye-tracking, to further be used collaboratively in games and VEs by playing or working together.

The increased use of eye-tracking in VEs and VR opens many opportunities for exploring complex multidimensional data sets. The combination of VR technology with eye-tracking, gesture, or body tracking will call for novel visualization techniques. However, there is a challenge in effectively visualizing eye-tracking data from one or many viewers in immersive VR environments. Here, novel ideas and techniques are needed from the visualization community to gain new insights and enhance understanding of the data. Effective solutions also need to be incorporated in future accessible and easy-to-use visualization and analysis tools. Only then can the visualizations be used by people not only in a research setting. As described earlier, the area of eSports could be such an application to enhance further. Developments with novel approaches in the immersive technologies industry could also feed back into data visualization.

Increased usage provides more opportunities for novel visualization techniques to surface, and reporting and classifying them is crucial to understanding how the field can develop. There is an earlier survey from 2017 (Blascheck et al., 2017) summarizing 2D/3D eye-tracking visualization techniques up the point of 2017 in a taxonomy. The rationale for this state-of-the-art systematic literature review is to describe established and emerging 3D eye-tracking visualization techniques for researchers and developers interested in understanding visual attention focus in VEs. The period for the search carried out in this paper corresponds to the last 5 years, starting from 2017 until the 17th of May 2022. The objective of the systematic review is to contribute to the knowledge of 3D visualization techniques of eye-tracking data in 3D scenes.

The remainder of the paper is organized as follows: Section 2 introduces eye-tracking foundations and some common 2D gaze visualization techniques. Section 3 briefly summarizes relevant previous work reviewing 3D eye-tracking visualization techniques in particular and highlighting seminal work. Next, Section 4 describes the systematic literature review method. This is followed by Section 5 which highlights the main results and recent research and developments in the area of 3D visualization techniques for eye-tracking data. The results are further discussed in Section 6, with a focus on possibilities, challenges, and future trends of visualization techniques for 3D VEs. Finally, Section 7 concludes the work and highlights directions of future research that are being unveiled by new technological developments.

## 2. Foundations of Eye-Tracking and 2D Visualization Techniques

Eye-tracking devices capture the gaze direction, also referred to as the Line of Sight (LOS) in 3D or the Point of Regard (POR) in 2D as a point on a plane (Sundstedt, 2012). The Point of Fixation (POF) is also used as a term when describing the gaze target in 3D space (Lappi, 2015).

Several eye-tracking techniques have been developed and the most commonly used in the last decade is called video-based eye-tracking (Duchowski, 2017). Video-based eye-tracking relies on video images of one or both eyes and an infra-red light emitter pointed at the user's eyes to compute the eye's orientation in space, hence the gaze direction of the user. To measure the POR, two features are extracted: the center of the pupil and the reflection of the infra-red light on the cornea. The corneal reflections are also referred to as Purkinje images (Crane, 1994; Duchowski, 2017). The positions of the corneal reflections relative to the pupil's center indicates where the user is looking at. Three types of hardware setups are currently available for video-based eye-tracking: eye-trackers that can be mounted on a screen, e.g., Tobii Pro X2-30, eye-trackers integrated into HMDs, e.g., Tobii Pro VR Integration^1^, or mobile headset eye-tracking (like glasses), e.g., Pupil Invisible^2^.

The human visual system utilizes different types of eye movements that can be divided into two main categories: stabilizing movements and saccadic movements (Lukander, 2003). Stabilizing movements consist of smooth pursuits, vestibular ocular reflex movements, optokinetic reflex movements, and fixations; they are used to keep an image still on the retina. Saccades and vergence movements are part of the second category, and they are responsible for repositioning the fovea, the area at the center of the retina having the highest visual acuity (Lukander, 2003; Sundstedt, 2012).

During a recording session, the eye-tracker collects a large amount of raw eye movement data at a high frequency, e.g., the Tobii Pro Fusion screen-based eye-tracker can reach a data sampling frequency of up to 250. Hz^3^ These raw data are processed to extract for example fixations and saccadic movements between fixations (Salvucci and Goldberg, 2000). To analyze the data, different visualization techniques are used. Blascheck et al. (2017) categorize them in two main classes: point-based, such as scanpaths or heat maps, and based on areas of interest.

### 2.1. Scanpath or Gaze Plots

**Figure 1:**
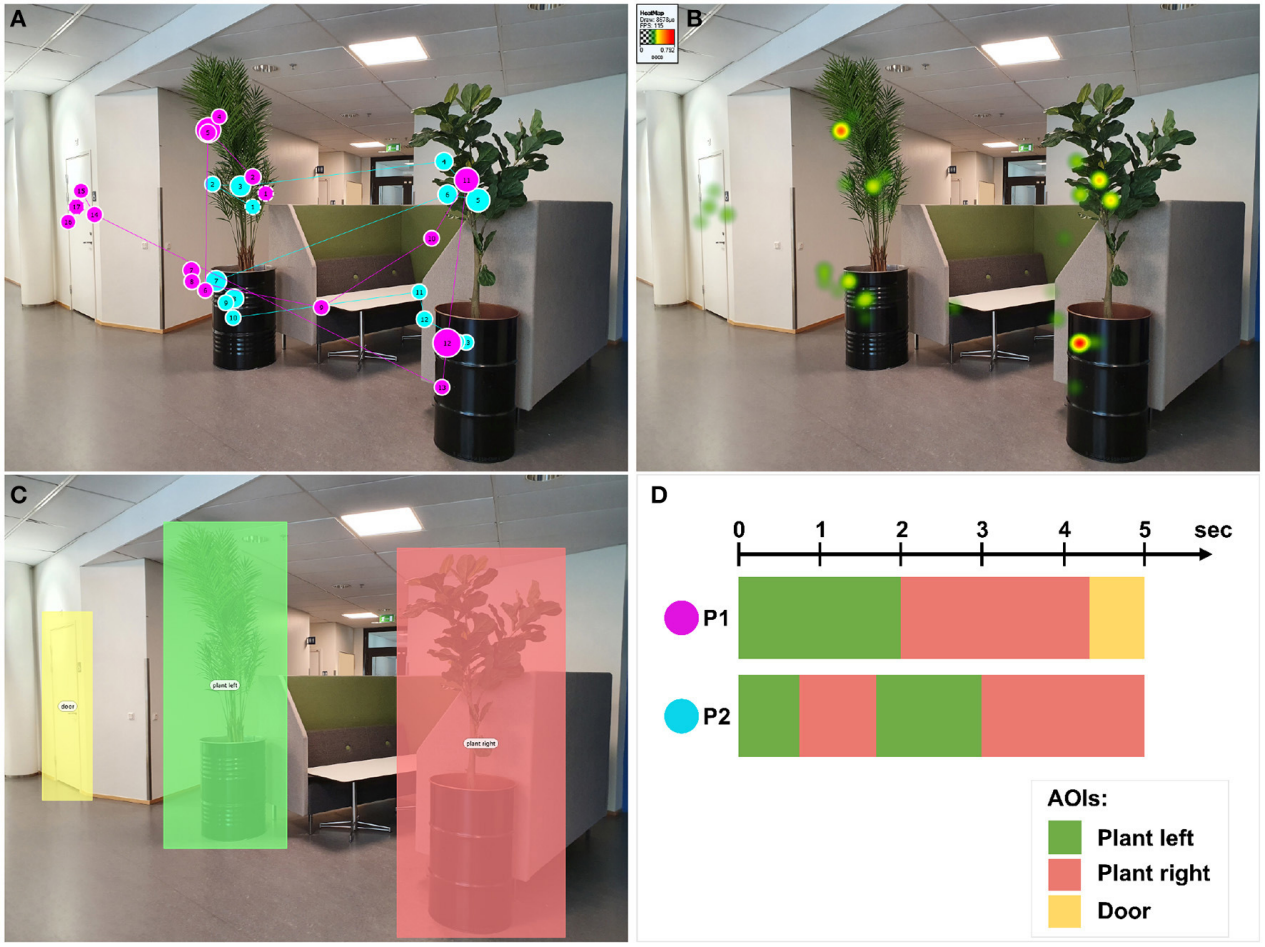
Examples of 2D gaze data visualization techniques. **(A)** Top-left: scanpaths of two participants (P1 in magenta and P2 in cyan) visualized with Tobii Studio software. **(B)** Top-right: heat map of the two participants showing the aggregated fixations duration, visualized with Tobii Studio software. **(C)** Bottom-left: definition of three areas of interest (AOIs). **(D)** Bottom-right: Schematic representation of a scarfplot visualization for the three pre-defined AOIs.

A scanpath, also called gaze plot, represents fixation points as circles on the top of the stimuli. The circle radius is proportional to the duration of the corresponding fixation. Two sequential fixation circles are connected by a line representing the saccadic movement going from the first fixation to the second one. In doing so, it is possible to create a full path of fixations. In each circle, a number can be shown describing the order of the fixations. This temporal information is useful, for example, to visualize which area of the stimulus has been focused first during a session. A scanpath represents the eye movement data of a specific user, and in an eye-tracking session with more than one user, all scanpaths are superimposed on the stimuli. In the case of several participants, this type of visualization could be confusing due to too many superimposed plots. A slightly different visualization can be used instead, called bee swarm, showing where all the participants were looking at the same time (Sundstedt, 2012). An example of scanpaths of two participants is shown in Figure 1A.

### 2.2. Heat Maps or Attentional Maps

A heat map, also called attentional map, represents an aggregation over time of gaze data, e.g., the number of fixations or the duration of the fixations of a user, usually using a color-based scale (e.g., green-yellow-red). This color-coded information is superimposed on the original stimulus and can be accumulated from all the participants. In order to avoid a scattered visualization and have a smooth map, a Gaussian filter is applied to the fixation areas (Duchowski, 2017). An example of heat map representing fixation duration is shown in Figure 1B.

### 2.3. Areas of Interest

The definition of areas of interest (AOIs) in the stimulus supports the analysis of specific regions that can be defined either manually by the analyst, for example, by selecting a rectangular or polygonal area of the stimulus (Figure 1C), or automatically. The automatic definition of AOIs can be done at the gaze data level, e.g., by performing clustering of the fixation locations, or by processing the stimulus, for example, by processing semantic segmentation of the stimulus. The most common visualizations based on AOIs show the variation of attention during the time between different AOIs, e.g., scarf plots (Richardson and Dale, 2005), or the relationship between them, e.g., transition matrix (Goldberg and Kotval, 1999). A schematic representation of the scarfplot visualization is shown in Figure 1D.

## 3. Previous Work on 3D Eye-Tracking Visualization Techniques

This section briefly presents the previous state-of-the-art in 3D visualization techniques of eye-tracking data and connects these with the described foundations. An extensive earlier survey (Blascheck et al., 2017) summarizes 2D and 3D eye-tracking visualization techniques up to 2017 and proposes a taxonomy of previous works according to categories based on the type of gaze data, the type of visualizations, and the nature of the stimulus. The authors identify four different aspects with which they classify the type of visualizations; (1) animated vs. static, (2) 2D vs. 3D, (3) interactive vs. non-interactive, and (4) in context vs. not in-context, i.e., methods including or not including the stimuli into the visualization. The survey reports 11 works focusing on 3D stimuli, seven of which present visualizations in context with the stimuli.

For point-based visualizations, representing the spatial and temporal distribution of eye movements (Blascheck et al., 2017), several works presented a 3D version of scanpaths rendered in the 3D environment of the stimuli. For example, Duchowski et al. (2002) present a method for analysis of gaze data from binocular eye-tracking systems in VR. To visualize the fixations and saccadic movements within the 3D scene, they render yellow spheres connected by yellow lines, where the spheres represent the fixations with a radius proportional to the fixation duration. Similar approaches have been used by Stellmach et al. (2010) and Pfeiffer (2012). In Pfeiffer (2012) the fixations are represented by blue spheres with radius indicating the high acuity visual angle around the optical axis, and cylinders represent saccades. Stellmach et al. (2010) propose two variants of 3D scanpath, one similar to the previously cited works using spheres representing fixation points, and a second one using conical representation encoding a larger amount of information compared to the spherical shapes: the apex of the cone representing the gaze position, the fixation duration can be encoded by the size of the base of the cone, the direction of the main axis of the cone represents the viewing direction of the observer, while their distance can be encoded by the height of the cone. Ramloll et al. (2004) propose a design of an integrated scanpath visualization for a particular subset of 3D stimuli: 3D objects with a simple geometry that can easily be flattened, e.g., a 3D model of a car. The main idea is to map the fixations on the surface of the object at the polygon level, and after the mapping, the stimuli is flattened in a 2D representation so that both 3D stimuli and the superimposed scanpath can be analyzed in 2D at a glance, having no occlusions.

Another natural integration of the gaze data in the 3D space of the stimuli is the projection of heat maps (or attentional maps) on the 3D stimuli. Stellmach et al. (2010) propose three types of heat maps: projected, object-based, and surface-based, with the last two being 3D representations fully combined with the 3D stimuli. The surface-based 3D heat map projects the gaze data directly on the surface of the 3D model; it is distributed over the surface using a gaussian filter, and its polygons are colored according to a colormap. Maurus et al. (2014) analyze a more complex 3D scene as stimuli, not only single 3D objects, and proposed a more accurate projection of the fixation data over the 3D geometry taking into consideration also the occlusions that each 3D element of the scene produces from the point of view of the observer. In Paletta et al. (2013), fixation hits with the 3D geometry are projected onto a 3D model with colormap with increasing values going through white, yellow, and red. Attentional maps have been proposed not only in terms of surfaces but also volumes. Pfeiffer (2012) presents a 3D attention volume in which each fixation point in 3D, instead of being illustrated by a sphere, is represented by a 3D Gaussian distribution on the 3D space of the stimuli centered on the 3D points of regard and encoded with a green-yellow-red colormap.

**Figure 2:**
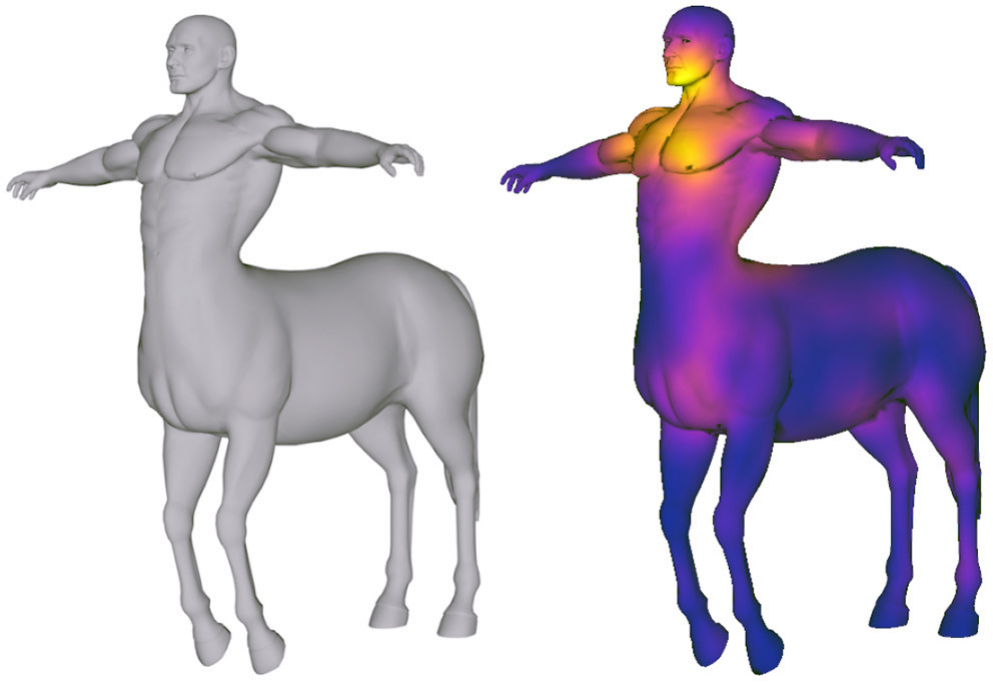
Example of a 3D surface-based attentional map (or heat map). The centaur mesh is part of the TOSCA high-resolution dataset (Bronstein et al., 2008).

AOI-based visualizations (Blascheck et al., 2017) have also been proposed for 3D stimuli. In Stellmach et al. (2010), the AOI is defined as a specific 3D object of the scene, and the object-based attentional maps proposed in this work assign a unique color to the whole surface of the 3D object, encoding with a single value the saliency of the whole 3D object with respect to the other objects in the scene. The chosen colormap is the same as the surface-based attentional map (green-yellow-red colormap). Hence the object that results to be more salient is visualized in red while the one with the lowest visual attention is in green. In Baldauf et al. (2010), a mobile eye-tracker device acquires both gaze data and video stream of a urban scene. To analyze the gaze data in context with the stimuli, a digital replica of the scene is available, and the gaze visualization consists of a gaze ray starting from the observer's location to the target model hit by the gaze. When the gaze ray points at a specific object, it changes its appearance by having a different color. A similar approach is shown in Pfeiffer et al. (2016), where the object hit by the 3D gaze ray is highlighted in green. One example of a 3D surface-based attentional map is shown in Figure 2, using a technique presented in Garro and Sundstedt (2020).

## 4. Methods

**Figure 3:**
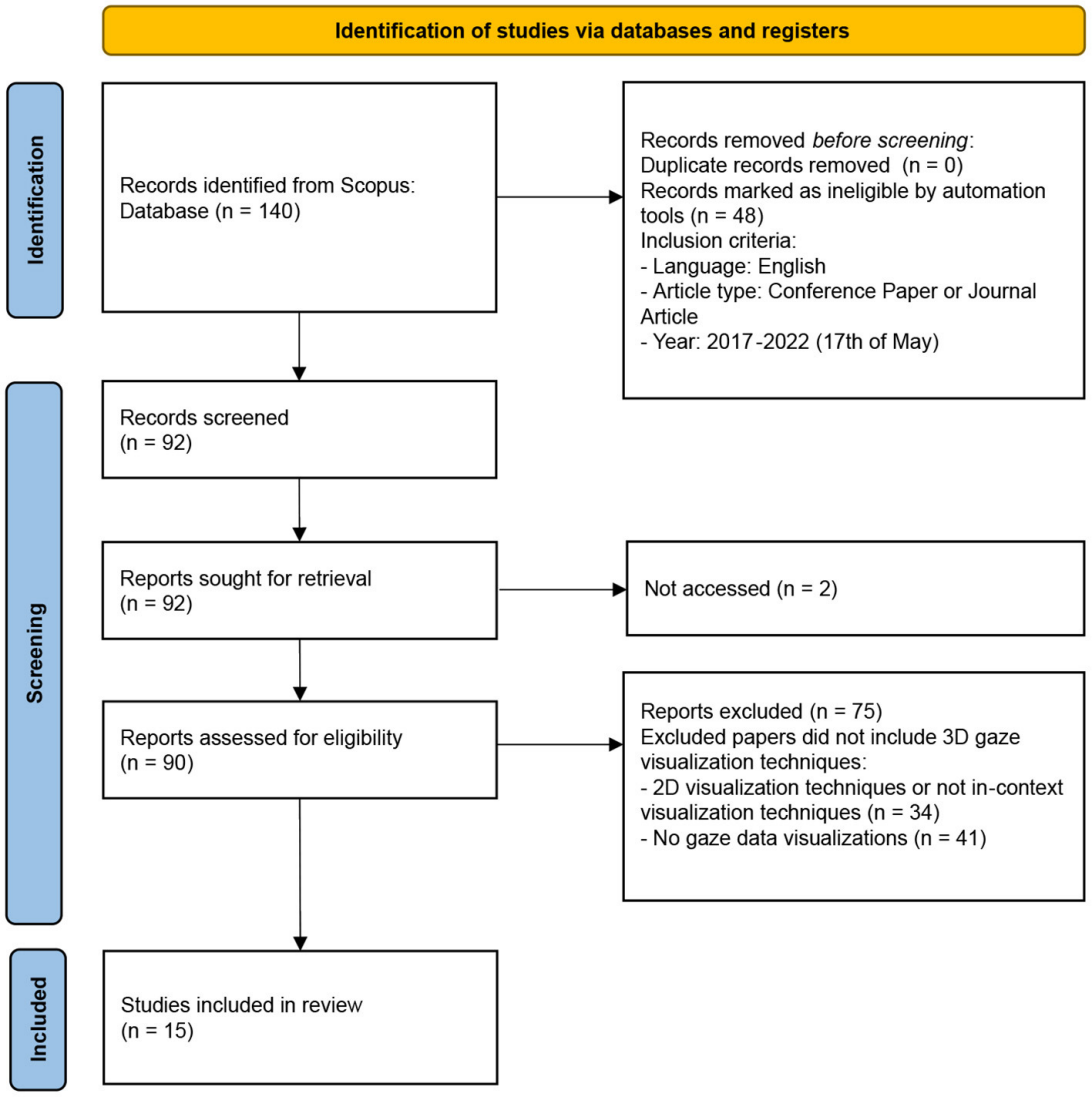
PRISMA 2020 flow diagram for systematic reviews (Page et al., 2021).

Figure 3 shows the PRISMA 2020 flow diagram for the systematic review and the applied eligibility criteria. The proposed systematic literature review has been performed using the information source Scopus, one of the largest databases of peer-reviewed literature, such as scientific journals, books, and conference proceedings. Scopus contains example publishers such as Elsevier, Springer, Association for Computing Machinery, and Eurographics. Scopus has also previously been reported to provide high-quality indexing of computer science publications (Cavacini, 2014).

We have focused on the publications published after the survey done by Blascheck et al. (2017) to cover the most recent work done on the topic. Hence, the range of publication years taken into consideration for the analysis was set to 2017–2022 (accessed 17th of May 2022). The search was done using the title, abstract, and keywords. This has the potential limitation of restricting the number of records found, however, if the full text had been used, the total number of retrieved publications had been too large (3654). The first search string used can be seen below:

- TITLE-ABS-KEY ((“eye tracking” OR “gaze”) AND (“visualization” OR “visualisation”) AND “3D”) AND (PUBYEAR > 2016))

This resulted in 140 publications, as shown in Figure 3. The search was then refined to have the language set to English and the document types limited to conferences and articles. This resulted in the final search string below:

- TITLE-ABS-KEY ((“eye tracking” OR “gaze”) AND (“visualization” OR “visualisation”) AND “3D”) AND (PUBYEAR > 2016) AND (LIMIT-TO (DOCTYPE, “ar”) OR LIMIT-TO (DOCTYPE, “cp”)) AND (LIMIT-TO (LANGUAGE, “English”))

The final literature search strategy resulted in 92 papers. The key to inclusion in the analysis was that the paper should have some 3D gaze data visualization technique, representing eye-tracking data in context with the 3D stimuli or a 3D representation of the stimuli. Two papers were excluded from the analysis due to not being accessible, as shown in Figure 3. One author initially screened all the 92 records. Some of these records were further discussed between both authors after the initial screening process to clarify their content and relevance. Papers focusing on purely traditional 2D eye-tracking visualization techniques, such as scanpaths or gaze plots, heat maps or attentional maps, or AOIs, were excluded from the review. Papers that presented a visualization of eye-tracking data not in-context with the stimuli were also excluded since they did not show fixation data directly in 3D scenes. These two groups together corresponded to 34 papers.

The remaining 41 excluded papers were deemed out of scope by not clearly visualizing gaze data. For example, some of these dealt with gaze interaction. They are still relevant in the context of eye-tracking interaction in VR, but that was beyond the scope of this review. As shown in Figure 3, the selection process resulted in 15 papers being included in the review. These were divided to be assessed by both authors independently and then discussed together. In the data collection process, both authors summarized the main research of the paper, including particular eye-tracking technology, observed stimuli, application context, type of 3D gaze visualization techniques, and opportunities and challenges.

## 5. Results

This section reports on the outcomes of the recent developments in the area of 3D eye-tracking visualization techniques, filling the gap between 2017 and 2022 (17th of May). Table 1 lists the identified included papers resulting from the literature search, categorized based on the eye-tracking technology, the type of stimuli observed, and the context of the proposed application.

**Table 1:** Description of papers chronologically ordered, based on eye-tracking technology, types of stimuli, and application context.

**References**	**Eye-tracking technology**	**Observed stimuli**	**Application context**
Ugwitz et al. (2022)	HTC Vive Pro Eye (HMD)	3D VE (VR)	Indoor evacuation behavior analysis
Jogeshwar and Pelz (2021)	Pupil Invisible (glasses)	Real environment	Room scene inspection
Breen et al. (2021)	Tobii 4C (screen-based) FOVE (HMD)	3D VE (screen) 3D VR	Gaze analysis during job interview simulator
Jogeshwar (2020)	Pupil Invisible (glasses)	Real environment	Room scene inspection
Shi et al. (2020)	Tobii 4C (screen-based) Oculus Rift CV1 + emb. eye-tracker (HMD)	2D map and 3D VE (screen) 3D VE (VR)	Indoor building inspection
Li et al. (2020)	Tobii Pro Glasses 2 (glasses)	Real environment	Room scene inspection
Rahman et al. (2020)	HTC Vive Pro Eye (HMD)	3D VE (VR)	Gaze analysis during educational experience
Bianconi et al. (2019)	HTC Vive + aGlass lenses (glasses)	3D VE (VR)	Interior architecture exploration
Singh et al. (2018)	SMI ETG 2 (glasses)	Real environment	Outdoor scene exploration
Hagihara et al. (2018)	Tobii Pro Glasses 2 (glasses)	Real environment	Room scene inspection
Naour and Bresciani (2017)	SR Research EyeLink 1000 Plus (screen-based)	2D video (screen) 3D VE (screen)	Real and virtual character inspection in the sports domain
Herman et al. (2017)	SMI RED250/Eye Tribe (screen-based)	3D object (screen)	Terrain data (GIS) analysis
Wang et al. (2017)	Pupil Eye-Tracker (headset)	Real object	Real objects inspection
Ma et al. (2017)	Eye Tribe (screen-based)	3D volumetric image (screen)	Volumetric dataset analysis
Song et al. (2017)	Tobii X60 (screen-based)	2D/3D volumetric image (screen)	Medical imaging analysis

Analyzing the type of stimuli observed in the retrieved papers, we can identify two main categories: real stimuli and digital stimuli. The real environment stimuli can be either single objects or more complex scenes (indoor or outdoor) captured by eye-tracking glasses or headsets. The digital stimuli are complex virtual environments, single 3D objects, and volumetrical medical images. While 3D objects and medical images have been shown *via* traditional screens, virtual environments have also been observed in VR using HMDs. However, some papers have also compared 2D techniques to 3D solutions in VEs being displayed on a traditional screen or in an HMD. Stimuli, including images or videos shown on a traditional computer screen, are referred to as 2D in Table 1. If a VE is being displayed on a traditional screen, this is referred to as 3D VE (screen) in Table 1. Finally, 3D stimuli being experienced in an immersive HMD are named 3D VE (VR).

Examples of eye-trackers used with traditional screens were the Tobii 4C, SR Research EyeLink 1000 Plus, SMI RED 250 device, EyeTribe eye-tracker, and Tobii X60 eye. Eye-tracking glasses include Pupil Invisible Eye-Tracker from Pupil Labs, Tobii Pro Glass 2 from Tobii Pro, and the SMI ETG 2 eye-tracker. The Pupil Eye-Tracker is mobile and head-worn, similar to a pair of glasses. Eye-tracker examples incorporated in HMDs include the HTC Vive Pro Eye and the Fove HMD. The HMD category also includes separate custom-made lenses for eye-tracking, which can be incorporated into a common VR headset, such as the HTC Vive, e.g., the 7invensum infrared lenses, also called A-Glass.

Regarding application contexts used for 3D gaze visualization analysis, it varies as shown in Table 1. Several of the papers have explored room scene inspections in general, whereas some have studied specific user behavior. Examples here include indoor evacuation, indoor building inspection or interior architecture exploration, gaze analysis during a job interview simulator, an educational experience, or for character movement in sports. Some work has also looked at outdoor scene exploration, terrain data (GIS) analysis, or real object inspection. Finally, a couple of papers were set in the medical domain, using eye-tracking in volumetric image analysis.

### 5.1. Gaze in 3D

Based on the analysis carried out identifying what 3D visualization techniques have been used in the papers, the outcome is clustered into three main categories: (1) gaze in real environments, (2) gaze in virtual environments, and (3) gaze on volumetric datasets. In the continuation of this subsection, we will describe the retrieved papers clustered by these types of stimuli.

#### 5.1.1. Gaze in Real Environment

Recent advancements in mobile head-mounted solutions for eye-tracking have opened the possibility of acquiring gaze data on egocentric camera video streams for analyzing the visual behavior of a user in a real environment carrying on everyday activities. Moreover, computer vision research on 3D reconstruction techniques from images or videos such as Structure from Motion (SfM) photogrammetry (e.g., Schönberger and Frahm, 2016), and Simultaneous Localization and Mapping (SLAM) (e.g., Mur-Artal et al., 2015) have developed and strengthen in the latest decades providing accurate and reliable digital reconstructions of real environments in terms of 3D point clouds or 3D meshes. The combination of 3D reconstruction techniques and mobile eye-tracking output seems a natural step for advancements in 3D gaze data analysis. In fact, several works have been recently presented proposing solutions for 3D mapping of egocentric gaze data over a 3D environment reconstructed from real indoor or outdoor scenes.

Hagihara et al. (2018) propose a system that processes gaze data from eye-tracking glasses and performs the 3D mapping of these data with respect to a 3D point cloud reconstruction of the environment. During an offline preparation phase, the physical scene is captured by a RGB-D camera and reconstructed using Visual SFM (Wu et al., 2011), an open source SfM software. Moreover, the system automatically segments 3D objects of interest in the scene using the method proposed by Tateno et al. (2015) that relies also on the depth info from the RGB-D camera. During the visualization phase, the user looks at the workspace with eye-tracking glasses which capture both the first person video (FPV) and 2D gaze points. The eye-tracker trajectory, hence the user position, is tracked in the reconstructed 3D scene, while the 2D gaze data is projected using a pinhole camera model into the 3D environment creating a 3D gaze. The proposed system has some similarities with Pfeiffer et al. (2016), however Hagihara et al. (2018) present a markerless solution for the user position tracking and uses an automatic segmentation of the objects of interest. The visualization of the 3D gaze data is integrated into the 3D scene, and a heat map is superimposed on the 3D objects of interest encoding the collision frequency of the 3D gaze ray with the specific object. The authors also adopt an object-based heat map assigning a color to each object, indicating its visual attention ranking.

A similar approach has been proposed by Singh et al. (2018) but, in this case, applied to real large-scale outdoor environments. The 3D reconstruction of the scene has been obtained with photogrammetry software (Agisoft PhotoScan) using as input the camera frames captured from the mobile eye-tracker (SMI ETG 2) together with some additional high-resolution photos. The output of the 3D reconstruction phase is a 3D mesh. To visualize the 3D gaze data, a ray-tracing technique is adopted using a spotlight model simulating the maximum visual acuity value centered on the gaze point and which decreases following a Gaussian distribution when moving peripherally. The authors proposed the application of two alternative gaze directions. The first one is based on the user's head position, which assumes that the user is always looking straight ahead, and the second one uses the actual gaze data acquired by the eye-tracker. In this work, different versions of 3D heat maps have been implemented to visualize the gaze data, mapping visual attention values directly onto the 3D mesh surface.

The approach proposed by Li et al. (2020) relies only on the camera frames captured by the wearable eye-tracker to reconstruct the real scene. The advantage of this solution is that there is no need to acquire the real environment with additional devices such as RGB-D cameras or 3D scanners, saving both time and money. SfM software COLMAP (Schönberger and Frahm, 2016) and OpenMVS^4^ multiview stereo reconstruction library have been used to reconstruct an indoor environment by obtaining a 3D mesh model and computing the camera position and orientation of each frame. The 3D user's line of sight is then computed with respect to the 3D model of the scene by estimating the line starting from the camera center and passing through the gaze location on the camera frame. Using image registration methods, multiple users' 3D gaze fixations can be visualized on the same 3D model of the scene. Similar to the previous works, the gaze data are visualized as 3D heat maps over the surface of the 3D model scene by counting the number of intersections between mesh triangles and the user's lines of sight.

Jogeshwar and Pelz (2021) present a pipeline called *GazeEnViz4D* to visualize gaze data in real indoor scenes in four dimensions, i.e., three spatial dimensions and time. An earlier work of this tool (Jogeshwar, 2020) is also included in the list of the retrieved papers. As the first room environment in Jogeshwar (2020), a rectangular room has been measured and modeled in 3D, and images of the room have been projected on the walls, called *wall templates*. The camera position and orientation of each video frame acquired by the eye-tracker (Pupil Invisible glasses) are computed *via* feature matching and the Perspective-n-Points (PnP) algorithm, and their 3D locations are displayed as points on the 3D model. The gaze points are represented as circles projected over the *wall templates*. In the extended version of the work (Jogeshwar and Pelz, 2021), the real scene is reconstructed using COLMAP, obtaining a 3D point cloud model. The geometry of the scene is not only limited to the walls of the room, being able to include more complex 3D objects. In Jogeshwar and Pelz (2021), the novel 4D gaze analyzer included in the pipeline allows for interactive analysis over time, as well as using arbitrary viewpoints that can be selected by the analyst. This tool also allows the analyst to zoom in, play/pause, speed up/slow down, and forward/reverse the eye-tracking data in a 3D context over time like a video. The 3D gaze points are computed by retrieving the 3D points in the point cloud corresponding to the feature point nearest to the 2D gaze point. This approach introduces limitations in the accuracy of the gaze point since it is directly related to the density of features extracted in each frame. The 3D gaze points are then visualized with a color-coded heat map generated from the 3D gaze points and overlaid to the neighbor 3D points following a Gaussian kernel distribution.

The works described up to this point deal with the representation of gaze data in real complex environments composed of different objects. On the other hand, the work by Wang et al. (2017) focuses on the inspection of a single object. Wang et al. (2017) present a system for accurate 3D gaze tracking on single physical 3D stimuli (3D printed objects) using a head-mounted monocular eye-tracker. The main idea is to relate the gaze direction with the geometry of the physical environment, which is known due to the presence of fiducial markers both on the 3D printed object and the surrounding environment. The paper includes an exploratory experiment to validate the accuracy of the proposed method on a 3D printed Stanford bunny. The task of the participants was to focus on each target marker applied to the 3D stimulus. To visualize the gaze positions obtained during the experiment, the authors choose a heat map on the surface of the digital 3D stimulus, i.e., the Stanford bunny 3D model. Since gaze visualization is not the main focus of this work, no further details are presented. However, from the figures, one could deduce that the chosen colormap is a linear “red to yellow” colormap. It is worth mentioning that the visualization of gaze direction using a heat map over the 3D stimuli has also been used in several other works on visual attention on 3D shapes, e.g., Wang et al., 2016, 2018; Lavoue et al., 2018; Alexiou et al., 2019; Garro and Sundstedt, 2020.

#### 5.1.2. Gaze in Virtual Environments

In Naour and Bresciani (2017), a visualization tool and two experiments were presented that explored one or multiple observers' viewing behavior when watching character animation movements. The chosen use cases were set in the sports domain (gymnastics and penalty kick). Their tool was designed for both non-experts and expert users and can visualize spatial and temporal gaze data. The aim of this work was to better understand human-body gestures. The material to be analyzed could either be a 2D video or 3D VR stimuli. In the first case, the user would manually annotate, and in the second case, motion capture was used. The output of their visualization tool is a colored mesh, which could be seen as a 3D heat map. Their algorithm builds the heat map from previously defined character joints and the concept of skinning.

Early work by Herman et al. (2017) proposed *3DgazeR*, a tool to analyze eye-tracking data of interactive single 3D objects, mainly tested with terrain 3D models, i.e., digital elevation models (DEM). The main functionality of the tool is converting the 2D screen coordinates of the gaze data gathered from the eye-tracker to the 3D coordinates of the model scene. Knowing the position and orientation of the camera with respect to the 3D scene, the tool performs a ray casting of the gaze 2D coordinates to the 3D model. 3DgazeR provides several different 3D visualizations of the 3D gaze data: (1) 3D raw data as points on the 3D surface, varying size, color, and transparency; 3D scanpaths, and 3D projected attention maps (Stellmach et al., 2010) as heat map projected on the 3D surface.

Bianconi et al. (2019) present a VR simulation exploring the legibility of multiple spaces in an indoor architectural scenario. The authors propose a case study in which an office building was modeled in 3D and visualized in an immersive VR experience by the participants through HTC Vive HMD with additional eye-tracking aGlass lenses. A group of eight people had to perform a series of wayfinding and orientation tasks in the VE without having prior knowledge about the building. Wayfinding was evaluated by exploring the user's orientation and movements. Based on the results from the task and eye-tracking data gathered, the design of the building was modified, and the same users performed the tests again. The gaze data were represented by heat maps overlaid on detection planes placed in proximity of core building elements such as walls, the floor, and points of interest. A qualitative analysis of the heat maps showed what parts of the office space the participants were looking at during the experience or if they noticed specific elements (e.g., signposting).

Another work in the context of building inspection in VR has been presented by Shi et al. (2020). In particular, the aim of the authors was to explore the relationship between visual attention and the development of spatial memory using three different display methods: a 2D drawing of the map of a real building, an interactive 3D model of the building shown on a screen, and a 3D model experience in immersive VR (Oculus Rift CV1 with an embedded eye-tracker). The participants got to inspect the building with one of the three display techniques while memorizing details and layout. After the review phase, they had to walk to the real building and explore it, trying to find discrepancies introduced in the digital versions. They did report on a strong positive relationship between visual attention and spatial memory development and that this was affected by the type of display techniques. The overall spatial memory development was improved in the 3D and VR compared to the 2D group. For the 3D model on screen and in VR, the 3D gaze data have been computed *via* raycasting and visualized as points in the VE together with a playback of the gaze movements.

Rahman et al. (2020) explore gaze data supporting a VR-based education scenario. Eye-tracking data can help provide real-time information to see if students are confused or distracted by looking at objects not relevant to the educational aim. In this study, the students were immersed in an outdoor VE of a solar field. Students' gaze data are shown to the teacher using six different visualization techniques. The proposed techniques were: (1) gaze ring, (2) gaze disk, (3) gaze arrow, (4) gaze trail, (5) gaze trail with arrows, and finally, (6) gaze heat map. The first three techniques took the gaze point into account, whereas the last three exploited gaze data over time. The presented techniques were evaluated in a within-subject user study to explore which one was the most effective in evaluating in real-time if students were distracted. All techniques were reported to be able to be used for single or multiple users, for example coloring the data for each person individually. Their initial results showed that gaze visualization techniques with the included time span, like a trail, were promising. This was followed by the gaze ring and gaze disk techniques. They also found that 3D heat maps for visualization over time or gaze arrows were not as promising. An interesting aspect of this work is that the visualization is proposed to be for real-time analysis. In this way, it could help teachers directly notice if students have a decrease in visual focus.

Breen et al. (2021) implemented a virtual job interview simulator gathering and visualizing different types of data, e.g., heart rate and electrodermal activity data, to detect stress levels and eye tacking to explore visual attention patterns of autistic individuals during simulated job interview scenarios. The VE consists of an office room with an interviewer avatar sitting at a desk. The main purpose of the simulator is to provide information supporting personnel for a better understanding of behavioral patterns of autistic individuals, for example, by studying eye contact and identifying what is causing stress. The VE of the simulated job interview can be experienced either by looking at a screen or in immersive VR (FOVE HMD). In relation to the eye-tracking data, the paper presents both 2D and 3D visualizations of the gaze data. In particular, the 3D gaze data are available for the VR experience and are computed *via* raycasting, i.e., finding the collision point of the gaze ray with the objects of the VE scene. The system offers four different variations of point-based visualization of 3D gaze data.

A recent work by Ugwitz et al. (2022) presents a workflow and software architecture for handling eye-tracking in complex interactive VEs experienced in immersive VR. The proposed workflow takes into account the whole pipeline, from the setup of the VE, to how to collect, correct and aggregate data, as well as the 3D data visualization. For the presented case study, they have modeled a VE of a building for analysis of indoor evacuation behavior. As some works mentioned above, the basic computation of 3D gaze data is done by raycasting the gaze ray into the geometry of the VE. Moreover, they implemented an algorithm to manage transparent objects or objects with see-through texture. The system uses multilevel *colliders* around 3D meshes as a 3D equivalent of 2D AOI. The 3D gaze points are visualized in an aggregated 3D heatmap composed of spheres located at the 3D gaze position and colored according to the number of other 3D gaze points nearby.

#### 5.1.3. Gaze on Volumetric Datasets

Volumetric datasets belong to the category of digital stimuli; however, due to the specific nature of the data, we describe in a separate subsection the papers that deal with the visualization of gaze data examining this type of stimuli. Volumetric images obtained, e.g., from computed tomography (CT) or Magnetic Resonance Imaging (MRI) scans, are three-dimensional scalar fields with a regular grid structure that can be visualized either partially (e.g., slicing in 2D images) or entirely (e.g., volume rendering) (Telea, 2014).

Ma et al. (2017) present a method to compute 3D salient regions on volumetric images. The 3D saliency volume is generated by processing multiple 2D saliency maps acquired from different angles. The gaze data are collected at image space while the user looks on a screen at a volumetric image that rotates at a regular pace, e.g., 12 degrees every 4 s. The 3D saliency map is created by back projecting the 2D saliency maps to the 3D volume. The visualization of the saliency values at the voxel level is encoded by color.

Song et al. (2017) propose *GazeDx*, a visual analytics framework for gaze pattern comparison of multiple users applied to volumetric medical images. Amongst several different 2D visualizations of gaze data, e.g., 2D scatterplot matrices, interactive temporal charts, *GazeDx* also includes a 3D visualization called *spatial view* in which the gaze points are superimposed on the 3D volume rendering image. To compute 3D gaze points from the raw gaze data, the authors adopted the *gaze field* approach presented by Song et al. (2014). The 3D gaze density is represented by a scalar volume with the same resolution as the stimulus. The gaze field is rendered upon the volumetric data by ray-casting encoding the voxel value with color and opacity. A 2D version of the spatial view is also included showing 2D gaze data superimposed on 2D multiplanar reformation images. The user can analyze and explore the 3D volumetric image enhanced with the 3D gaze points rotating the 3D volume. The framework also provides filtering operations based on the segmentation of anatomical parts. The authors evaluated the *GazeDx* framework with two case studies where radiologist experts used the tool and performed a gaze analysis of seven colleagues, each reading two patients' computed tomography(CT) images.

### 5.2. 3D Gaze Visualization Techniques

This section presents the 3D gaze visualization techniques shown in the analyzed papers in more detail. We have identified three main clusters: 3D position-based techniques, 3D heat map techniques, and volumetric heat map techniques. The 3D position-based category includes all visualizations representing the specific gaze position in the 3D environment in different ways, i.e., where the gaze ray hits the geometry of the 3D environment. 3D heat maps visualizations represent an aggregation of 3D gaze data within the time from one or several users. Finally, volumetric heat maps also display aggregated gaze data adapted to volumetric datasets. Table 2 shows the identified 3D gaze visualization techniques found in the included papers.

**Table 2:** 3D visualization techniques used in the analyzed papers. The papers are clustered per stimuli type: real environment (RE), virtual environment (VE), and volumetric dataset (VD).

**Stimuli**	**References**	**3D Position-Based Techniques**	**3D Heat Maps**	**Volumetric Heat Maps**
RE	Jogeshwar and Pelz, 2021	Gaze position (ring)	Heat map (point cloud)	
		Gaze ray		
	Jogeshwar, 2020	Gaze position (ring on 2D walls)		
	Li et al., 2020		Surface heat map (mesh)	
	Singh et al., 2018		Surface heat map (mesh)	
	Hagihara et al., 2018		Surface heat map (mesh)	
			Object heat map	
	Wang et al., 2017		Surface heat map (mesh)	
VE	Ugwitz et al., 2022	Gaze position (sphere)	Heat map (spheres)	
	Breen et al., 2021	Gaze position (flattened, sphere)		
	Shi et al., 2020	Gaze position (point, no detail)		
		Gaze movement		
	Rahman et al., 2020	Gaze position (ring, disk, arrow, trail, trail/arrow)	Surface heat map (mesh)	
	Bianconi et al., 2019		Surface heat map (mesh)	
	Naour and Bresciani, 2017		Surface heat map (mesh)	
	Herman et al., 2017	Gaze position (spheres)	Surface heat map (mesh)	
		Scanpaths	Surface heat map (mesh)	
VD	Ma et al., 2017			3D saliency volume
	Song et al., 2017			Gaze field

In addition to visualizing gaze data in 3D, several papers also combined this information with a trajectory showing how the user moved through the environment. Ugwitz et al. (2022) visualize user movement and interaction and make their *pathvisualizer* code available on GitHub. Bianconi et al. (2019) and Jogeshwar (2020) also visualize the path taken in 3D by the user as an addition over gaze data using lines and points, respectively. Shi et al. (2020) show user walking trajectories using red lines. Finally, Jogeshwar and Pelz (2021) visualize the observer's location in the environment using red dots.

#### 5.2.1. 3D Position-Based Techniques

The analyzed papers present several different visualizations representing the specific gaze position in the 3D environment.

##### 5.2.1.1. Gaze Points/Spheres

In the *3DgazeR* tool by Herman et al. (2017), several different visualization methods were used. First, they plotted the raw data using points on a 3D surface and varied the size, color, and transparency. For example, they mapped female raw data to the color red and male raw data to the color blue. Shi et al. (2020) visualize the gaze point in red in the 3D scene, but they provide no further specific details on its shape in the text. Breen et al. (2021) use four different point-based visualizations. One technique is a playback of a single green 3D sphere representing the 3D gaze data (raycasting of gaze ray to the VE geometry) moving over time. The other visualization options are variations allowing the user to plot all fixations after each other or at once. If the user wants to see the current gaze point at the same time as the complete set, it is shown in a contrasting color to the rest. Breen et al. (2021) also use the *scanpath* terminology, but even though it is possible to explore the gaze order in real-time, the points have no connecting lines between them like in traditional scanpath visualizations for 2D. Ugwitz et al. (2022) visualize gaze movement with a small white sphere object at the fixation point.

##### 5.2.1.2. Gaze Ring

A colored ring displayed at the gaze location, to visually highlight the gaze point in the virtual environment, is used by Rahman et al. (2020) who also introduced the terminology *gaze ring*. Other works also apply this visualization in 3D reconstructions of real environments Jogeshwar (2020); Jogeshwar and Pelz (2021).

##### 5.2.1.3. Gaze Disk

Rahman et al. (2020) also introduced the concept of a *gaze disk* to visually indicate the gaze point in the 3D environment. They argue that the disk would be smaller than their gaze ring and therefore less intrusive.

##### 5.2.1.4. Gaze Arrow

Rahman et al. (2020) also used 3D arrows to indicate the gaze with the arrow tip at the gaze point. In their experiment, each arrow was represented to be 3 m long, pointing to distance objects (around 30 m) away from the observer. The arrows were mapped to different solid colors, like red, green, blue, yellow, and turquoise.

##### 5.2.1.5. Gaze Trail

One technique introduced by Rahman et al. (2020) is what they refer to as a *gaze trail*, which makes gaze analysis possible over time and at the same time being able to see the most current gaze point. The trail was created using a system with particle emitters that moved along the gaze points. Each particle was displayed for 3 s, and then newer particles were shown brighter than older ones. This is a technique they refer to as being designed for VR rather than desktop or mobile scenarios. Ugwitz et al. (2022) also used a kind of trailing fade-out of the last few previous eye-tracking coordinates.

##### 5.2.1.6. Gaze Trail/Arrow

Rahman et al. (2020) also introduced a *gaze trail with arrows* as a new technique for VR. This technique is reported to be similar to the gaze trail, but it has static line segments instead of the particle emitter system. They use a minimum length segment and plots one arrow per three line segments. A smaller sphere is used here to show the current gaze point, and the arrow indicates the gaze movement direction. The movement history did not fade to highlight the potential distractions in their studies with students' attention focus. Hence, their technique shows both the current gaze and past information simultaneously.

##### 5.2.1.7. 3D Scanpaths

Similar to scanpaths in 2D, several previous works have also adopted the concept in 3D, as mentioned in Section 3. In the analyzed papers, only Herman et al. (2017) applies this technique by displaying semi-transparent 3D spheres proportional in size to the fixation duration. A transparency attribute has been added to the spheres to deal with possible occlusions. Saccadic eye movements were then shown as these 3D fixation points but with lines connecting the spheres. They also used a value to control transparency to deal with overlapping data.

#### 5.2.2. 3D Heat Maps

There are multiple works that have used some form of solution based on heat maps in 3D. From the included papers, we have classified these into four categories: (1) 3D surface heat maps, (2) point cloud heat maps, (3) spheres heat maps, and (4) object heat maps. They all provide the aim to visualize aggregated gaze data, which are mapped to different color schemes to show a lower or higher concentration of visual focus.

##### 5.2.2.1. 3D Surface Heat Maps

In Naour and Bresciani (2017), the eye gaze data is mapped to character joints rather than the mesh directly. The joint weights then influence the color of the mesh, similar to skinning, to create a heat map on the 3D character where blue shows no attention and red is mapped to the highest. Herman et al. (2017) use 3D projected attention maps, similar to Stellmach et al. (2010). Here the heat map is projected onto the 3D surface and encoded with a custom colormap. Wang et al. (2017) also used a heat map on the 3D stimulus, the Stanford bunny model, in their experiment. The gaze visualization was not the focus of the work, but their figures show that the heat map was visualized using a linear color map from red to yellow.

Hagihara et al. (2018) generated a heat map on top of the observed 3D objects in the scene. They used as a basis a collision frequency detection between a gaze ray and the object in the scene to infer input to the heat map.

Singh et al. (2018) generated several different surface-based 3D heat maps. They also used ray-casting from the points of gaze to the 3D model, sampling with a Gaussian drop-off in terms of visual acuity. They used ray tracing to simulate a spotlight drop-off and checked for hits with 3D mesh triangles. They visualize gaze density on the 3D models, comparing a head-centric or gaze-centric approach. They also use colored heat maps on the 3D models, as well as acuity-based heat maps or surface-based heat maps, as in Stellmach et al. (2010).

Bianconi et al. (2019) used heat maps projected onto detection planes in the proximity of walls, floors, and points of interest in their modeled VE. The colors chosen in their heat map were quite common, with red representing more visual focus and blue less.

Li et al. (2020) also checked gaze points for intersection with the triangles on the 3D mesh. They then proposed to color the triangles. As an example, more hits would be colored red. However, they suggest that purely coloring could look discrete and hence not ideal. Instead, they also propose that it is better to visualize the results using a heat map for an improved result. They use a diffusion filter, which has a similar effect as a Gaussian filter, to smooth the data. In their example, areas with more attention will receive red colors, whereas less observed areas would be colored green.

In addition to their 3D gaze visualization techniques displaying historic gaze data, Rahman et al. (2020) developed a gaze heat map for VR with a cooling effect to show that the attention was being reduced. The opacity and saturation vary with the density of the nearby gaze and the age of the gaze points. Here the gaze locations were rendered on the object using a custom shader. With the cooling effect, they were able to compare it with their techniques on gaze trail and gaze trail with arrows described earlier. They also support multiple observers by showing a color for each user.

##### 5.2.2.2. Point Cloud Heat Maps

In Jogeshwar and Pelz (2021), 3D gaze points are estimated from 2D gaze points by finding key points (generated by the algorithm for each image used to create the environmental model) nearest to the 2D gaze point in the point cloud. A Gaussian kernel is then scaled to the uncertainty in gaze estimation, which is overlaid on every 3D gaze point closest to the key feature to its respective 2D gaze point, generating a heat map. In this work, the gaze ray is also visualized from the observer to points in the environment.

##### 5.2.2.3. Spheres Heat Maps

In the work by Ugwitz et al. (2022) the *heatmapvisualizer*, they do not use a classical colored heat map, but rather color gaze points represented as spheres using a gradient. In their example, spheres in 3D which are focused on more will receive a red color, whereas lower concentrations of fixations will result in gray spheres.

##### 5.2.2.4. Object Heat Maps

Similar to Stellmach et al. (2010), the work by Hagihara et al. (2018) also include a visualization at the object-level of the ranking of visual attention for the objects of interest segmented from the 3D mesh reconstruction of the real scenes. They colored the objects in the scene in a complete color depending on the number of hits they received. Red in their color selection relates to a high number of hits and blue to a low number of hits.

#### 5.2.3. Volumetric Heat Maps

Ma et al. (2017) generated a volumetric representation of the saliency map superimposed on a 3D volumetric image and 3D saliency volume. Here a colormap based on the red channel was used, and for the 3D saliency volume, it was visualized using DVR with a colormap. The colors chosen for the colormap were low to high values mapped to blue, green, yellow, and red. Another heat map technique in this category was introduced by Song et al. (2017), which used a volumetric representation of the 3D gaze density superimposed on the 3D volume rendering image.

## 6. Discussion

There are many aspects that lead to a large amount of data being produced during eye-tracking sessions. In order to analyze gaze behavior in 3D environments, one also needs to be able to make sense of what the eye movements are being focused on. For example, Ugwitz et al. (2022) mention that all interactivity needs to be logged on its own and information about the motion of objects or the user in the scene itself. This is particularly important if one wants to make conclusions based on eye-tracking related to the user interaction in the VE. The user task also influences the complexity of the analysis. Shi et al. (2020) point out, though, that too much information could lead to cognitive overload.

An interesting trend is also to analyze complex data from multiple users, perhaps even looking at the same scene simultaneously or at different times. This further complicates the eye-tracking analysis and leads to more cluttered scenes which need to be visualized effectively. Ugwitz et al. (2022) also state that processing all data is time-consuming. They propose to use an interval subset of gaze data or process an area defined by a spatial polygon to simplify the analysis. Several of the papers also presented the benefits of being able to replay the gaze data (Jogeshwar and Pelz, 2021) or show it in real-time (Rahman et al., 2020).

In the process of mapping gaze positions to objects, it is important to be able to identify objects or parts of them in order to map fixations to meaningful data. This process can be simpler for VE with predefined virtual objects that fixation data can be mapped to Sundstedt et al. (2013). However, the distance of the user from the object also plays a role in the interpretation of the mapping, as reported by Ugwitz et al. (2022), and it needs to be taken into account implementing suitable AOI colliders. When eye-tracking real environments, this information might not be known at all, even if there are algorithms aiming to automatically classify objects in the scene (Tateno et al., 2015). In 2D scenes, the distance to the objects is known, and this makes easier the process of mapping gaze positions to scene content. In 3D, points in the scene have various depths, and techniques need to be adjusted to take this into account. Traditional techniques need to accommodate this as well as deal with temporal aspects in 3D scenes (Ugwitz et al., 2022). Blascheck et al. (2014) also mentioned that it could be problematic to map fixations back to 3D objects. The computation of gaze ray intersecting objects in 3D cannot always be used, e.g., in the visualization of a medical volumetric dataset, transparent exterior structures can still be visualized by direct volume rendering, but the user might be focusing on internal structures (Ma et al., 2017).

Jogeshwar and Pelz (2021) mentioned that a downside of traditional techniques and tools is that one is restricted to a single viewpoint and that the analysis can also be time-consuming to go through frame-by-frame. This problem could also exist in 3D if the camera is static. Allowing interactive viewpoints could provide better opportunities to see occluded objects or mitigate object distortions caused by being further away (Herman et al., 2017).

Another issue is being able to deal with complex VEs, such as those reconstructed from the real environment. The accuracy of the 3D reconstruction of real environments is key since it influences motion tracking and 3D gaze mapping, hence the visualization analysis. This is valid in particular in the case of 3D reconstruction of small objects being part of large scenes (Hagihara et al., 2018). Moreover, when using camera frames for the 3D reconstruction, issues related to motion blur can affect the accuracy of the 3D reconstruction (Jogeshwar, 2020) as well as the presence of large featureless regions in the scene (Li et al., 2020; Jogeshwar and Pelz, 2021).

However, they also point out benefits by stating that automatic methods have advantages over manual methods and that it is key that more complex scenarios and tasks can be explored. Jogeshwar (2020) also points out that SFM algorithms rely on the environment being static. The analysis of real scenes taking into account also moving elements could possibly be tackled by different types of algorithms specific for dynamic scene reconstruction (Ingale and Divya, 2021).

Regarding 2D vs. 3D visualizations, Rahman et al. (2020) state that traditional visualization techniques, such as line charts and scatter plots, are not ideal for visualizing gaze data in VE. They point out that 3D techniques are more suitable for VR. Jogeshwar and Pelz (2021) also state that it is hard to show gaze data for larger and more complex environments in 2D and for the user to get a good understanding of the overall scenario. In this regard, they argue that 3D has advantages over 2D stimuli. Shi et al. (2020) compared different media, from 2D drawings, 3D on screens, and 3D in VR HMD for task performance in building discrepancies tasks between the real environment and different visualization techniques. They found 3D to give better spatial knowledge and be more realistic. Bianconi et al. (2019) also reported the benefits of 3D by being able to use immersive 3D gaze analysis to explore the impact of various design choices, resulting in reduced virtual complexity. However, Breen et al. (2021) also point out that it can be useful to have both non-immersive 2D and VR for users that are not as comfortable with 3D. Singh et al. (2018) mention that there is a gap in what one can do in 2D and outdoor physical, real-world environments.

As we can see from this systematic literature review, there have been advancements in trying to fill this gap, but there are still open research challenges to deal with the complexity of the overall problem. Some of these problems are related to the large amount of data that needs to be handled. Another is related to the accuracy in reconstructions of real scenes. One important area is also the automatic classification of objects or AOIs in VEs. Finally, more work on the evaluation of suitable 2D vs. 3D gaze visualization techniques in immersive environments is needed, taking into account the scene complexity and task.

Based on the carried out review, some areas warrant future research and developments. One of these areas is to explore how to better deal with single and multiple users, such as in collaborative VEs using eye-tracking technology. Another area of interest is future HMDs or combinations of eye-tracking with additional sensors, like EEG, heart rate, and galvanic skin response. With more data gathered from users in immersive VR, more research on appropriate visualization techniques is also needed. Due to all these aspects, future eye-tracking analysis tools being incorporated into game engines or new immersive VR visualization software have these challenges to deal with. Ugwitz et al. (2022) also point out that visualization techniques might need to be incorporated in 3D engines. It is also essential to evaluate such tools and software with real users to aid in the potential complexity.

There is a risk that is making all data available to users simultaneously, even if possible, will result in more confusion. Hence, guidelines and opportunities to filter data for analysis seem crucial, which is also mentioned by Rahman et al. (2020), for larger user groups and in Ugwitz et al. (2022). There might also be different needs depending on whether the users are novices or experts. Li et al. (2020) highlight that there is considerable potential for applications related to evaluating user attention in large and complex environments. Shi et al. (2020) also propose personalized information systems adjusted to the user. Breen et al. (2021) propose to integrate 3D visualization in a web application directly and to provide critical insights and data without showing too much to avoid an overabundance of data. They say it is essential to balance raw data and clear insights and that different user roles might need to see different things. When working with eye-tracking data, it is also important to consider privacy issues and data storage (Rahman et al., 2020), in particular in health-related studies. The future solutions need to take a human-centered approach, allowing machine intelligence to help simplify the parts of the 3D visualization analysis in VEs where possible.

## 7. Conclusions and Future Work

This paper has presented a systematic literature review in the area of 3D visualization techniques and analysis tools for eye-tracking in 3D environments. This includes both natural environments being reconstructed for 3D analysis or VEs like simple 3D objects or more complex virtual scenes to be experienced in VR. Eye movement data can be a great asset to analyzing human behavior in 3D HMD VEs or 2D video from eye-tracking glasses. The huge potential for growth in VR applications such as games or simulations is evident. Due to an increase in the use of VR and mobile eye-tracking solutions, such as glasses, further 2D/3D visualization techniques are needed for analyzing VEs and 2D video content from eye-tracking glasses.

We argue that many important developments have happened in the last few years, including the advent of new technologies, an exponential increase in the volume of data being collected, and the massive adoption of mobile devices worldwide. Eye-tracking can also be combined with other sensors for multi-sensory interaction, and research is needed to evaluate the effectiveness of these combinations in VEs in the future. It is only in the last few years that sensors, such as eye-tracking and biofeedback, have started to appear in commercial video games and VR applications. Novel VR technology headsets, including more advanced eye-tracking solutions, for example, are also going to become further available to the mass market in the future, allowing us to develop timely and novel multi-sensory interaction techniques. With this forthcoming growth in data from VR applications, novel, effective visualization techniques are needed to gain new insights and enhance understanding of data that would not otherwise have been possible.

## Data Availability Statement

The original contributions presented in the study are included in the article, further inquiries can be directed to the corresponding author/s.

## Author Contributions

VS conceptualized the idea, performed the bibliographic search, and wrote the first draft of the manuscript. VG helped during the analysis of the retrieved studies and contributed in writing the first version of this manuscript. Both authors contributed to the revised manuscript, contributed to the article, and approved the submitted version.

## Funding

This work has been financed partly by KK-stiftelsen, Sweden, through the ViaTecH Synergy Project (contract 20170056).

## Conflict of Interest

The authors declare that the research was conducted in the absence of any commercial or financial relationships that could be construed as a potential conflict of interest.

## Publisher's Note

All claims expressed in this article are solely those of the authors and do not necessarily represent those of their affiliated organizations, or those of the publisher, the editors and the reviewers. Any product that may be evaluated in this article, or claim that may be made by its manufacturer, is not guaranteed or endorsed by the publisher.

## Footnotes

^1^ https://www.tobiipro.com/product-listing/vr-integration/

^2^ https://pupil-labs.com/products/invisible/

^3^ https://www.tobiipro.com/siteassets/tobii-pro/user-manuals/tobii-pro-fusion-user-manual.pdf/?v=2.4

^4^ https://github.com/cdcseacave/openMVS
